# Mild Traumatic Brain Injury Causes Nociceptive Sensitization through Spinal Chemokine Upregulation

**DOI:** 10.1038/s41598-019-55739-x

**Published:** 2019-12-20

**Authors:** Peyman Sahbaie, Karen-Amanda Irvine, De-Yong Liang, Xiaoyou Shi, J. David Clark

**Affiliations:** 1 Department of Anesthesia, Perioperative and Pain Medicine, Stanford University, School of Medicine, Stanford, CA 94305 USA; 2 Anesthesiology Service, Veterans Affairs Palo Alto Health Care System, 3801 Miranda Ave (112-A), Palo Alto, CA 94304 USA

**Keywords:** Chemokines, Diseases of the nervous system

## Abstract

High rates of acute and chronic pain are associated with traumatic brain injury (TBI), but mechanisms responsible for the association remain elusive. Recent data suggest dysregulated descending pain modulation circuitry could be involved. Based on these and other observations, we hypothesized that serotonin (5-HT)-dependent activation of spinal CXC Motif Chemokine Receptor 2 (CXCR2) may support TBI-related nociceptive sensitization in a mouse model of mild TBI (mTBI). We observed that systemic 5-HT depletion with p-chlorophenylalanine attenuated mechanical hypersensitivity seen after mTBI. Likewise, selective spinal 5-HT fiber depletion with 5,7-dihydroxytryptamine (5,7-DHT) reduced hypersensitivity after mTBI. Consistent with a role for spinal 5-HT_3_ serotonin receptors, intrathecal ondansetron administration after TBI dose-dependently attenuated nociceptive sensitization. Also, selective CXCR2 antagonist SCH527123 treatment attenuated mechanical hypersensitivity after mTBI. Furthermore, spinal CXCL1 and CXCL2 mRNA and protein levels were increased after mTBI as were GFAP and IBA-1 markers. Spinal 5,7-DHT application reduced both chemokine expression and glial activation. Our results suggest dual pathways for nociceptive sensitization after mTBI, direct 5-HT effect through 5-HT_3_ receptors and indirectly through upregulation of chemokine signaling. Designing novel clinical interventions against either the 5-HT_3_ mediated component or chemokine pathway may be beneficial in treating pain frequently seen in patients after mTBI.

## Introduction

Traumatic brain injury (TBI) has an annual incidence of 295 per 100,000 of the population and is a leading cause of trauma-related disability worldwide^1,2^. In the United States, more than 2.5 million injuries occur per year (Centers for Disease Control and Prevention, 2015), with the majority of these cases being mild in nature^3^. While many symptoms of mild TBI dissipate rapidly after injury, these patients frequently experience unusually high rates of acute and chronic pain^4,5^. Headache commonly occurs after TBI, although pain can be widespread and may include the back and extremities^6,7^. Mechanical allodynia and other sensory abnormalities are seen in the limbs of some TBI patients as demonstrated by quantitative sensory testing, consistent with neuroplastic changes in the brain and, possibly spinal cord^8^. Disrupted descending modulation of nociceptive signaling has been suggested to contribute to pain after TBI in patients^9,10^.

Both noradrenergic (inhibitory) and serotonergic (facilitatory and inhibitory) pathways are involved in descending modulation of nociception^11^. Damage to centers involved in the descending modulation of pain have been identified in animal models and human TBI^10,12^.

Previous work has characterized pain-related, cognitive and other behavioral outcomes in a mouse model of mild concussive TBI (mTBI) including loss of diffuse noxious inhibitory control (DNIC). Nociceptive sensitization after mTBI was refractory to conventional anti-inflammatory and anti-neuropathic analgesics in this model, consistent with other central pain syndromes^13^. Potentially helping to explain the results, studies by our lab and others have shown the CXC Motif Chemokine Receptor 2 (CXCR2) and its endogenous ligands (CXCL1-3, 5, 7) may regulate pain after TBI, surgery and nerve damage^14–16^. With respect to TBI, spinal CXCR2 appeared to be mostly expressed by lumbar dorsal horn neurons and was found to regulate peripheral nociceptive sensitization using the rat lateral fluid percussion (LFP) model of TBI^14^. Further studies demonstrated a strong association between the spinal dysregulated serotonin input, neuroinflammation and neuronal activation early on after LFP injury^17^. At this time, interactions between TBI-induced dysregulation of endogenous pain control circuits, neuroinflammation and CXCR2-dependent nociceptive sensitization after mTBI have not been identified.

We therefore hypothesized that activated descending serotonergic facilitation would be responsible for the enhanced spinal neuroinflammation, upregulated chemokine expression and nociceptive sensitization seen after TBI. This type of mechanism, if demonstrated to exist, might explain the pain and nociceptive hypersensitivity common in patients after TBI, and would offer avenues towards novel clinical treatments of this important TBI-related pain problem.

## Results

### Assessment of mild TBI (mTBI) induced mechanical hypersensitivity after systemic or spinal -5-HT depletion

**Figure 1:**
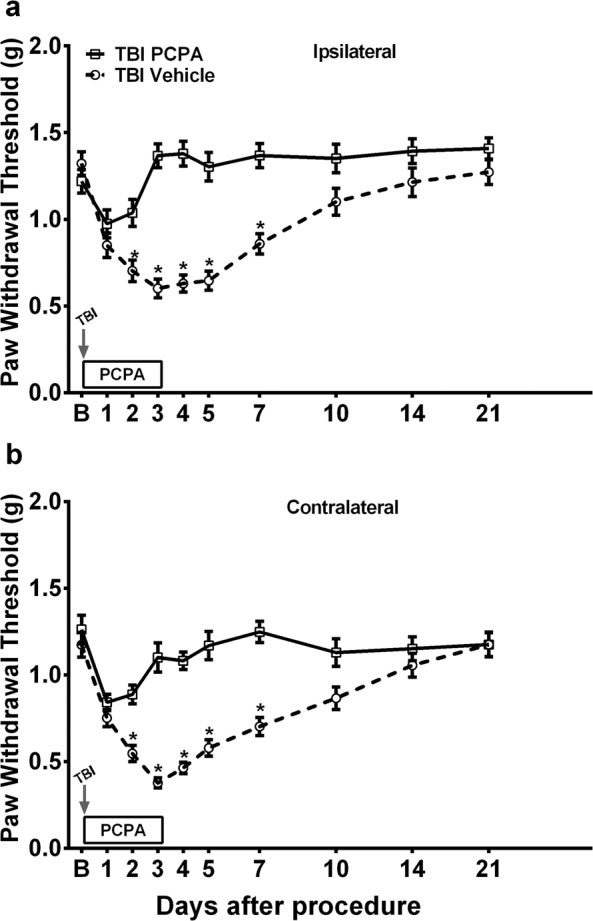
Assessment of mild TBI-induced mechanical hypersensitivity after systemic 5-HT depletion. Once daily systemic  treatment for 4 days with 100 mg/kg p-Chlorophenylalanine (PCPA) in mild TBI mice resulted in recovery of mechanical hypersensitivity in **(a)** ipsilateral and **(b)** contralateral hindpaw (relative to injury side). Arrow indicates when the TBI was performed. Data were analyzed by multiple t tests using the Holm-Sidak method to correct for multiple comparisons. ^*^Indicates significant difference in comparison with respective limb measurements in vehicle group. Error bars: SEM, n = 10/group.

**Figure 2:**
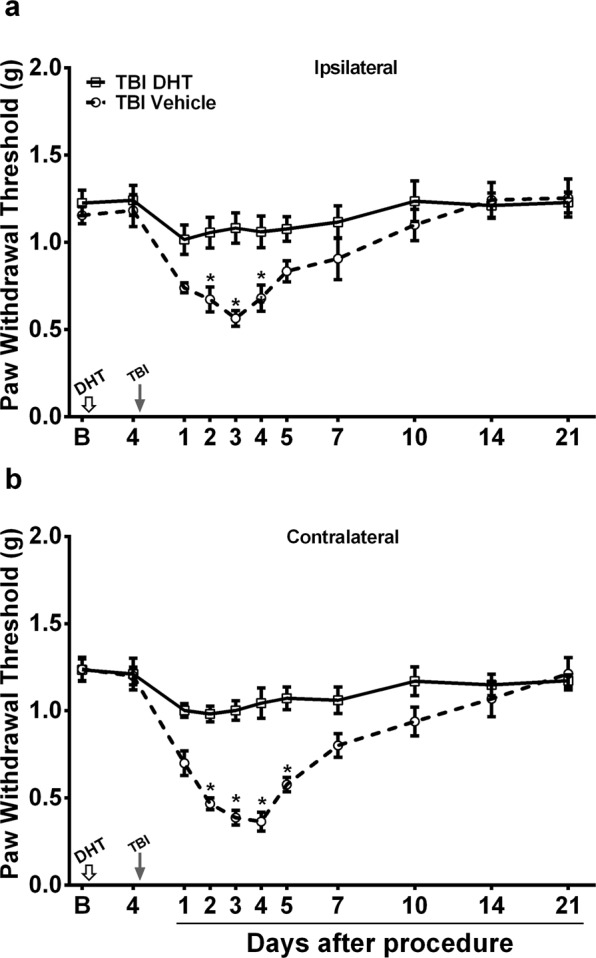
Assessment of mild TBI-induced mechanical hypersensitivity after spinal 5-HT depletion. Mice treated with intrathecal 5,7-DHT (Dihydroxytryptamine) displayed significantly reduced mechanical hypersensitivity relative to vehicle treatment after TBI in both **(a)** ipsilateral and **(b)** contralateral limbs (relative to injury side). Mice were pretreated with systemic desipramine hydrochloride (25 mg/kg, *i.p*.) 45-min prior to 5,7-DHT (50 µg, *i.t*.) administration (white arrow). TBI was performed 4 days after 5,7-DHT injection (black arrow). Data were analyzed by multiple t tests using the Holm-Sidak method to correct for multiple comparisons. ^*^Indicates significant difference in comparison with respective limb measurements in vehicle group. Error bars: SEM, n = 7–8/group.

We have previously shown an increase in hindpaw mechanical sensitivity after mTBI in mice with peak increases lasting for 72 h and a gradual recovery to baseline values by 14 days post-injury^13^. To assess the role of serotonin signaling in mTBI-induced mechanical hypersensitivity, systemic 5-hydroxytryptamine (5-HT) depletion was achieved with once daily treatment with p-Chlorophenylalanine (PCPA) for four days. The first dose was given immediately after mTBI. PCPA treatment has been shown to result in about 90% depletion of systemic and CNS (cortex, brainstem and spinal cord) 5-HT levels^18^. Within two days of initiating PCPA treatment, mTBI mice exhibited a gradual and lasting recovery of mechanical sensitivity of both ipsilateral and contralateral limbs to baseline values compared to vehicle treated mTBI mice. (Fig. 1a,b). Next, we confined the 5-HT depletion to serotoninergic neurons with lumbar spinal terminals in mTBI mice using 5, 7-dihydroxytryptamine (5, 7-DHT). The 5,7-DHT treatment has been shown to result in about an 85% reduction of 5-HT levels in lumbar spinal tissue within 4 days^17,19^. Mice that were pretreated with 5, 7-DHT displayed significantly reduced mechanical hypersensitivity relative to vehicle treatment after mTBI in both ipsilateral and contralateral limbs relative to injury side **(**Fig. 2a,b**)**. Treatment with vehicle, PCPA or 5, 7-DHT had no effect on basal paw withdrawal thresholds of sham mice at any time-point after mTBI (data not shown). As the mTBI-induced nociceptive changes were seen to be more pronounced in the contralateral limb relative to injury side, all subsequent evaluations were carried out contralaterally.

### Effects of the 5-HT_3_ receptor antagonist, ondansetron, on mTBI-induced mechanical hypersensitivity

**Figure 3:**
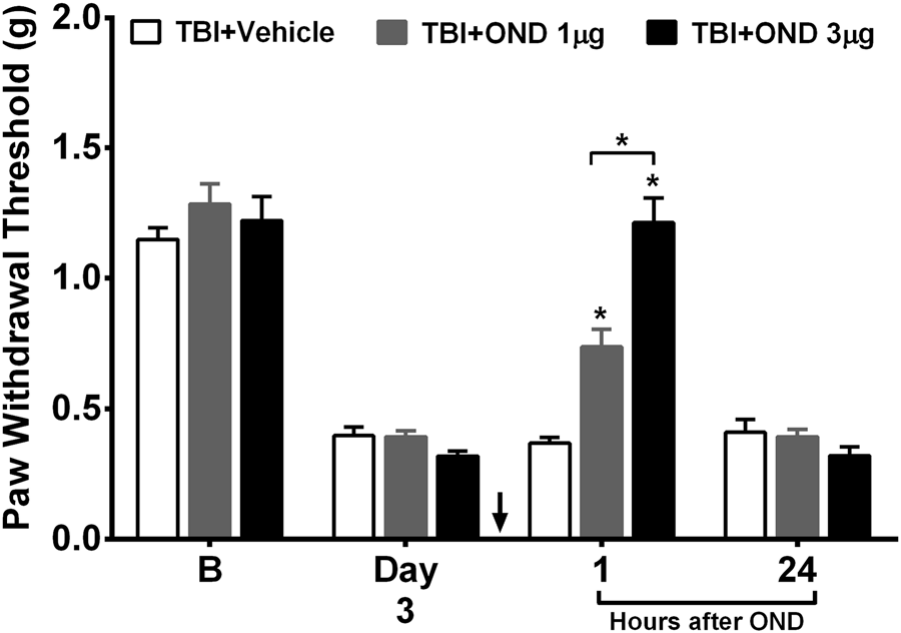
Effects of the 5-HT_3_R (serotonin receptor 3) antagonist, ondansetron, treatment on mild TBI-induced mechanical hypersensitivity. Intrathecal administration of ondansetron (black arrow) 1 and 3 µg on day 3 after TBI dose dependently attenuated nociceptive sensitization in separate groups of mice compared to vehicle treatment. Data were analyzed by multiple t tests using the Holm-Sidak method to correct for multiple comparisons. Error bars: SEM, n = 8/group, ^*^ indicates significant difference in comparison with vehicle group or between drug doses as indicated.

The 5-HT_3_ receptor (5-HT_3_R) is a ligand-gated ion channel, activation of which results in a rapid excitatory response in both the CNS and PNS. It has been implicated in mediating the pronociceptive actions of descending serotonergic spinal input after spinal cord and neuropathic injuries^20,21^. Therefore, the effects of the selective 5-HT_3_R antagonist ondansetron on mechanical hypersensitivity after mTBI were assessed. Ondansetron, which was intrathecally (*i.t*) administered on post mTBI day 3, dose dependently attenuated nociceptive sensitization with the 3 µg dose restoring mechanical thresholds to baseline values **(**Fig. 3**)**.

### Assessment of CXCR2 ligand expression levels after mTBI and spinal 5-HT depletion

Our lab has previously shown a role for spinal CXCR2 in nociceptive sensitization in a model of rat TBI^14^. We therefore wanted to determine whether CXCR2 played a similar role in mouse TBI. We also sought to extend the analysis to measurements of CXCR2 ligand expression (CXCL1-3, 5, 7) as these are up-regulated in some pain states^22^. Lumbar spinal cord tissue harvested at 3 days post-TBI revealed significant increases in mRNA levels of both the receptor, CXCR2, and two of the five CXCR2 ligands; CXCL1 and CXCL2 compared to uninjured mice **(**Table 1**)**. Analysis at 7 days post-TBI revealed a significant increase in the CXCL3 ligand in addition to persistently elevated CXCL1 and CXCL2 levels.

**Table 1:** Gene expression changes in the CXC chemokine ligands family and their receptor in the lumbar spinal cord after closed head mild TBI.

	Days after Injury		
	0	3	7
CXCL1	1.08 ± 0.16	2.74 ± 0.16^***^	2.42 ± 0.22^**^
CXCL2	1.09 ± 0.11	3.53 ± 0.67^***^	3.37 ± 0.61^***^
CXCl3	1.09 ± 0.12	2.00 ± 0.51	3.03 ± 0.37^***^
CXCL5	1.08 ± 0.18	1.93 ± 0.46	1.96 ± 0.24
CXCL7	1.08 ± 0.15	1.74 ± 0.35	1.82 ± 0.18
CXCR2	1.05 ± 0.09	2.02 ± 0.26^*^	1.91 ± 0.11

Data were analyzed by one-way ANOVA followed by Fisher’s LSD post-hoc tests and presented as mean ± S.E.M fold change relative to baseline, n = 6/group, *p < 0.05, **p < 0.01, ***p < 0.001 for comparison to baseline (Day 0).

**Figure 4:**
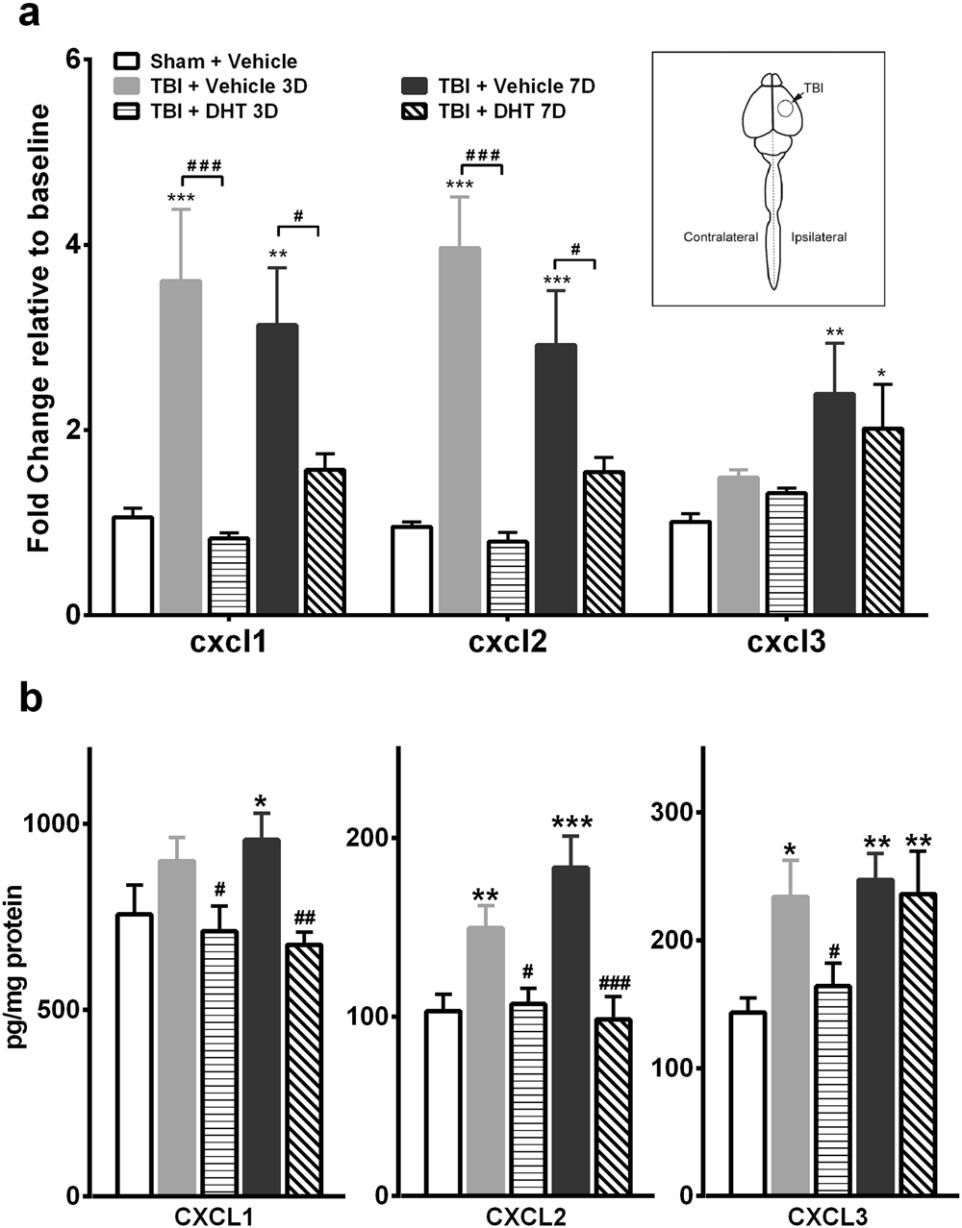
Assessment of CXCR2 (CXC Motif Chemokine Receptor 2) ligands mRNA and protein expression levels after mild TBI and spinal 5-HT depletion. Mice were pretreated with systemic desipramine hydrochloride (25 mg/kg, i.p.) 45-min prior to 5,7-DHT (50 µg, *i.t*.) administration. **(a)** Spinal application of 5,7-DHT attenuated the increased CXCL1 and CXCL2 mRNA expression levels on days 3 and 7 treatment after injury compared to vehicle group. **(b)** 5,7-DHT attenuated the increased CXCL1 and CXCL2 protein levels seen on days 3 and 7 treatment after injury, while lowering the elevated CXCL3 expression levels only on day 3 after TBI compared to vehicle treated TBI mice. Data were analyzed by one-way ANOVA followed by Fisher’s LSD post-hoc tests. Error bars: SEM, n = 6/group for mRNA and n = 8/group for protien levels messaurements, *p < 0.05, **p < 0.01, ***p < 0.001 for comparison to sham/vehicle group and ^#^p < 0.05, ^##^p < 0.01, ^###^p < 0.001 for comparison to respective TBI/vehicle groups.

We then investigated the effect of spinal 5-HT depletion on mTBI induced expression level changes of the CXCR2 ligands. Intrathecal administration of 5, 7-DHT significantly attenuated the increase of CXCL1 (Day 3 and 7: p < 0.001), and CXCL2 (Day 3: p < 0.001, day 7: p < 0.01) mRNA expression levels at both time points when compared to vehicle-treated mTBI mice **(**Fig. 4a**)**. No significant effect of 5-HT depletion was seen for the CXCL3 expression levels at any time point after mTBI compared to vehicle-treated mTBI mice.

Because mRNA expression and protein levels do not always change concordantly, protein levels of the above-mentioned ligands were measured using ELISA. Our results showed that protein changes in spinal cord tissue were similar to the mRNA findings after mTBI **(**Fig. 4b), and that 5,7-DHT treatment reduced mTBI-simulated CXCL1 and CXCL2 levels.

### Effects of CXCR2 antagonist treatment on mTBI induced mechanical hypersensitivity

**Figure 5:**
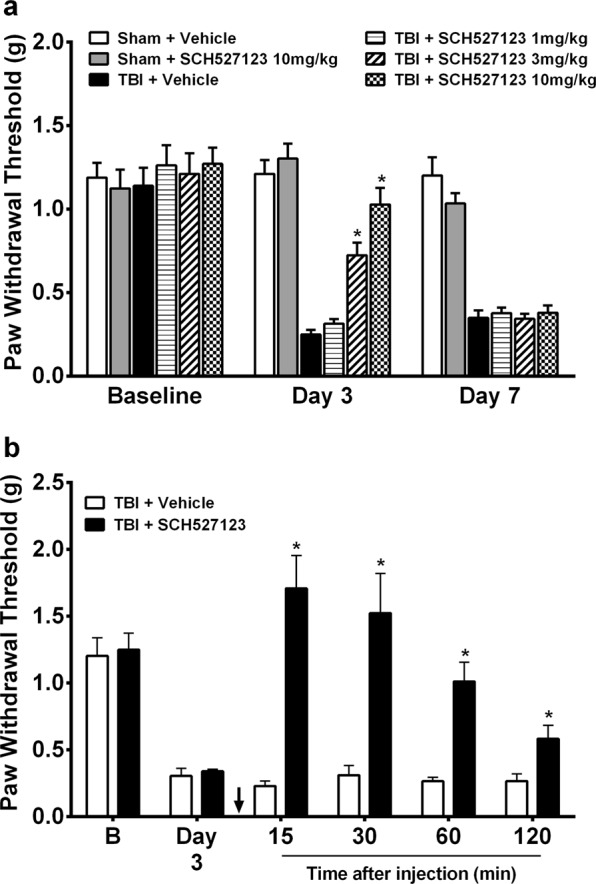
Effects of CXCR2 antagonist treatment on mild TBI induced mechanical hypersensitivity. **(a)** Systemic SCH527123 treatment for 7 days (Day 0 to 6) dose dependently attenuated mechanical hypersensitivity after TBI with the 10 mg/kg dose having the maximal reversal effect. Mechanical hypersensitivity after mild TBI remained attenuated during SCH527123 treatment and was reestablished upon cessation. **(b)** Intrathecal application of SCH527123 (black arrow) 3 µg on day 3 after mild TBI restored mechanical thresholds to baseline values. Data were analyzed by multiple t tests using the Holm-Sidak method to correct for multiple comparisons. ^*^Indicates significant difference in comparison with limb measurements in TBI/vehicle group. Error bars: SEM, n = 6–8/group.

The effects of systemic and spinal CXCR2 antagonist SCH527123 administration on mechanical withdrawal thresholds after mTBI were assessed, as CXCL1, 2 and 3 exert their effects by signaling through the chemokine receptor CXCR2^16^. Systemic SCH527123 treatment for 7 days (Days 0 to 6) dose dependently attenuated mechanical hypersensitivity after TBI with the 10 mg/kg dose having the maximal reversal effect **(**Fig. 5a). Mechanical hypersensitivity was reestablished within 24 hours of cessation of drug administration (Day 7). Spinal administration of SCH527123 on day 3 after TBI effectively restored mechanical thresholds to baseline values, highlighting the importance of spinal CXCR2 and its chemokine ligands in maintenance of nociceptive sensitization after mTBI **(**Fig. 5b**)**.

### Effects of 5-HT depletion, 5,7-DHT or CXCR2 antagonist, SCH527123 treatment on mTBI-induced neuroinflammation in lumbar superficial dorsal horn

**Figure 6:**
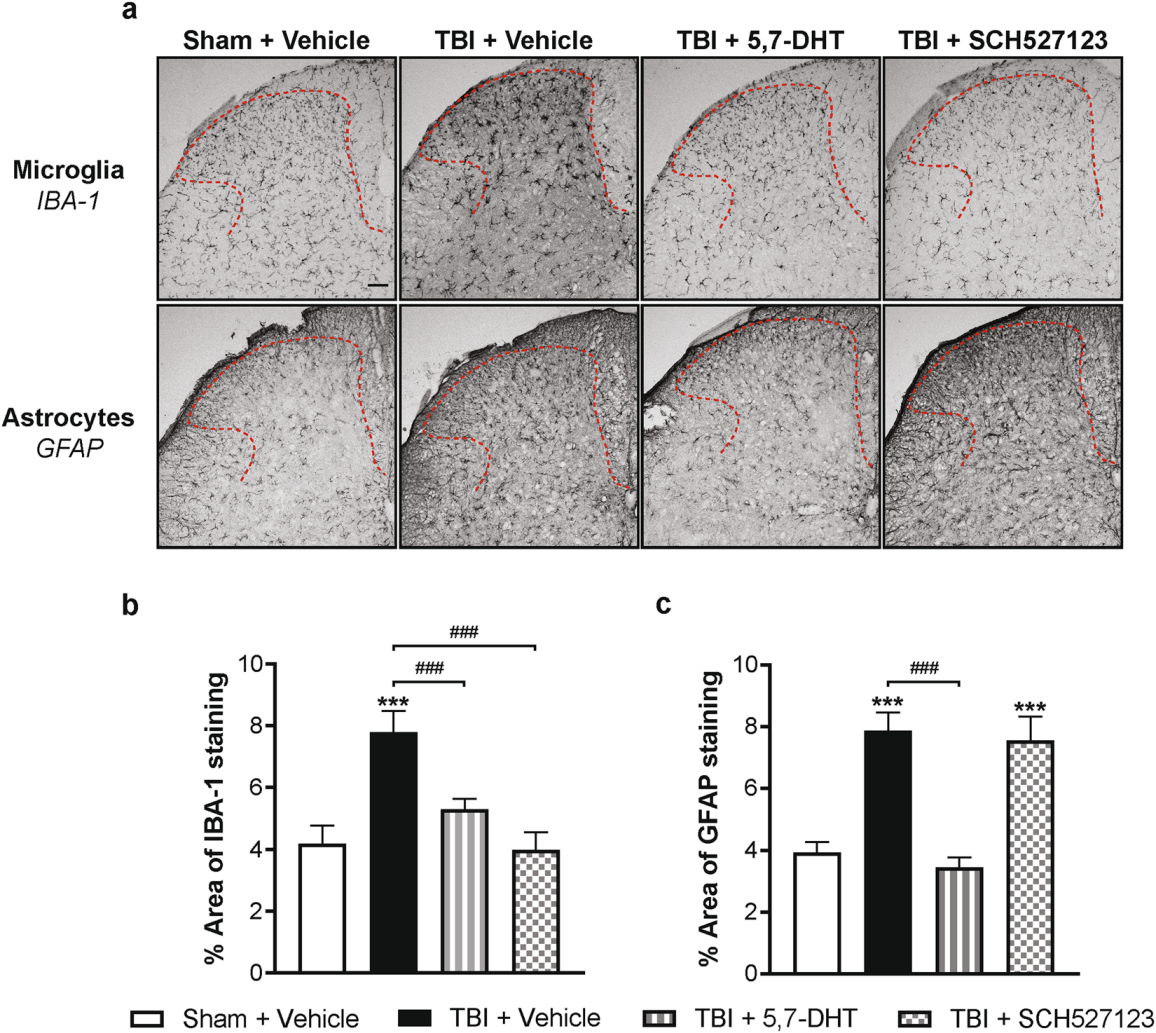
The effect of 5,7-dihydroxytryptamine (5,7-DHT) or the CXCR2 antagonist, SCH527123, on the TBI-induced neuroinflammation in superficial dorsal horn of the lumbar spinal cord. **(a)** Photomicrographs of the lumbar spinal dorsal horn (outlined in red) from sham, TBI vehicle-treated or 5,7-DHT-treated or SCH527123-treated mice stained with the microglial marker, IBA-1, (**a**: top panel) or the astrocyte marker, GFAP, (**a**: bottom panel). **(b)** TBI resulted in a significant increase in IBA-1 expression at 3 days post-injury (DPI) in vehicle-treated mTBI mice compared to vehicle-treated sham mice. The TBI-induced increase in IBA-1 expression was significantly reduced by both the spinal application of 5,7-DHT (50 µg, *i.t*.) and the systemic administration of SCH527123 (10 mg/kg, *i.p*). **(c)** A significant increase in GFAP expression in the superficial dorsal horns was observed on day 3 post injury and the spinal application of 5,7-DHT significantly attenuated this increase. In contrast, SCH527123 had no effect on the TBI-induced astrocyte response to injury. Data were analyzed by one-way ANOVA followed by Fisher’s LSD post-hoc tests. Error bars: SEM, n = 10–12/group, ***p < 0.001 for comparison to sham/vehicle group and ^###^p < 0.001 for comparison to respective TBI/vehicle groups. Scale bar: 50 µm.

At 3 days post-injury (DPI), the effect of mTBI, serotonin depletion or CXCR2 antagonist treatment on the astrocyte and microglia populations was assessed in the superficial dorsal horns of the lumbar spinal cord using GFAP and IBA-1 markers respectively (Fig. 6a**)**. mTBI resulted in a significant increase in IBA-1 expression in the dorsal horn compared to sham mice at 3 DPI (Fig. 6b, p < 0.001). The mTBI-induced increase in IBA-1 expression was significantly absent in mice that had spinal 5,7-DHT (50 µg, i.t.) or systemic SCH527123 (10 mg/kg, i.p) treatments (p < 0.001). Furthermore, 5-HT depletion attenuated the mTBI-induced increase in GFAP expression when compared to vehicle treated-mTBI mice (Fig. 6b, p < 0.001). In contrast, SCH527123 treatment had no effect on the mTBI-induced increase in GFAP expression in the dorsal horn.

## Discussion

Traumatic brain injury is a common injury associated with immediate and delayed sequelae, even when the initial injury is mild. While motor deficits, cognitive impairment and longer-term risks of neurodegenerative disease are well established consequences, pain is less often discussed despite its high prevalence^5^. Relatively little is known concerning the mechanism of pain after TBI, although several groups have succeeded in providing evidence of sensitization in laboratory models^12,14,23–26^. In these studies, we used a recently developed murine model of mild closed-head TBI (mTBI) to examine the role of descending serotonergic innervation in supporting pain after mTBI. Our main observations were: (1) Pain-related changes (mechanical nociceptive sensitization) occurred rapidly after m TBI in mice and lasted 1–2 weeks, (2) Disruption of serotonergic signaling using PCPA or intrathecal 5,7-DHT sharply reduced behavioral evidence of nociceptive sensitization as did blockade of spinal 5-HT_3_ receptors, (3) The expression of the CXCR2 receptor and its cognate ligands such as CXCL1 and CXCL2 in spinal tissue were up-regulated after mTBI but these effects were greatly limited in 5,7-DHT treated mTBI mice, (4) Spinal 5,7-DHT pretreatment attenuated acute neuroinflammation, as seen by decreased astrocyte and microglia upregulation accompanying mTBI, and (5) The selective CXCR2 antagonist SCH527123 potently reduced nociceptive sensitization in mTBI mice and was effective in reducing spinal microglial upregulation. Taken together, our observations suggest that mTBI causes dysregulation in descending serotonergic pain control systems that work through 5-HT_3_ receptors and the up-regulation of chemokine signaling to cause nociceptive sensitization after mTBI.

Serotonergic descending pain modulation can both facilitate and inhibit pain. The cell bodies of the principle neurons projecting to the sensory areas of the dorsal horn of the spinal cord are located within the rostroventromedial medulla (RVM)^11,27^. Augmented RVM serotonergic activity has been linked to many types of pain including pain after nerve injury^28^, in the setting of chronic inflammation^29^, and after the chronic administration of opioids^30^. The participation of the descending serotonergic fibers from this area of the brainstem in supporting pain after TBI has not received much focused study. However, MRI-based imaging techniques have suggested damage to brainstem nuclei such as the periaqueductal grey (PAG) and PAG-RVM communication after TBI^10,31^. Additional post-mortem data has demonstrated brainstem damage in patients after TBI, although the large amount of white matter and other anatomical considerations of the brainstem may afford it some protection during head trauma^32^.

Descending fibers from the RVM release serotonin onto neurons in the dorsal horn of the spinal cord causing either the facilitation of nociceptive signaling (5-HT_1_, 5-HT_3_), or inhibition of signaling (5-HT_2A_, 5-HT_7_)^11,27^. Although the effects of serotonin are complex, both agents we employed for the purpose of depleting serotonin (PCPA and 5,7-DHT) reduced TBI-induced sensitization without affecting thresholds in non-TBI animals, consistent with findings in other pain models^33,34^. Involvement of the spinal 5-HT_3_ receptor was demonstrated through intrathecal injection of ondansetron that provided dose-dependent effects. In fact, this receptor has been demonstrated in several other pain models including ones representing spinal cord injury^20^, peripheral nerve trauma^35^, inflammatory pain^36^, and others to support sensitization. Moreover, it should be noted that others have shown wide-spread dysfunction in the regulation of serotonin levels after TBI that might be related to depression after this form of injury. Some of this problem may be caused by diminished levels of serotonin transporter (SERT) expression^37^.

Additional studies conducted by our group and others suggest that TBI damages brainstem structures and that this damage may lead to changes in the spinal cord favoring nociceptive signal transmission. For example, a detailed neuropathological analysis of the rat brainstem 7 days after lateral fluid percussion (LFP) TBI demonstrated changes in the RVM and other brainstem pain regulatory centers^12^. Astrocytosis and microgliosis were observed in both the RVM and periaqueductal grey matter (PAG), indicative of neuroinflammation. There was also evidence of neuronal loss in the RVM of LFP rats. Moreover, a robust microglial response in the superficial dorsal horns of lumbar spinal cord was observed^17^. Additional data related to RVM dysfunction after TBI comes from post-mortem examination of TBI victims suggesting loss of neurons in the medullary serotonergic dorsal raphe nucleus^32^. Thus, our findings in the mouse closed head mTBI model complement existing observations.

While several lines of evidence suggest that dysfunctional serotonin signaling may be a root cause of pain at least in the early stages after TBI, the effects of serotonin in spinal cord tissue may not be limited to the immediate effects of serotonin exciting spinal dorsal horn neurons through 5-HT_3_ receptors. For example, both microgliosis and enhanced Fos expression, an indicator of neuronal activation, were seen in sensory processing areas of the spinal cord 7 days after LFP TBI injuries in rats^12^. Earlier work in TBI rats demonstrated elevations in pain-related molecules in spinal dorsal horn tissues such as brain-derived neurotrophic factor (BDNF) and the chemokine receptor CXCR2, with epigenetic mechanism controlling the increased expression of the latter^14,23,38^. Further observations have demonstrated an acute upregulation of CXCR2 signaling in brain parenchymal astrocytes after closed head TBI^39^. However, no mechanism integrating brainstem dysfunction with spinal changes was provided. Here, the findings from our experiments involving suppression of descending serotonergic influences using 5,7-DHT demonstrate that this input is required for not only the neuroinflammation enhancement of CXCR2 expression, but also the enhanced expression of endogenous ligands for CXCR2 including CXCL1 and CXCL2. Moreover, the effectiveness of the CXCR2 antagonist SCH527123 administered both systemically and intrathecally in our model was clearly demonstrated.

While the stimulation of spinal 5-HT3 receptor/ion channels can support nociception by directly supporting depolarization of the nerves on which the receptors are expressed, our data suggests that a second chemokine mediated cascade may be involved. In this regard it is notable that the interaction of descending 5-HT3 pronociceptive signaling and spinal chemokine production has been demonstrated in a model of peripheral inflammation. This study demonstrated nociceptive sensitization to be due to neuron-glia-neuron interactions mediated by 5-HT driven upregulation of neuronal chemokines including fractalkine^40^. The study directly demonstrated that this neuronal fractalkine drive supported microglia and astrocyte activation required for sensitization. In the case of our work, we studied a central injury rather than a peripheral one. Thus no nociceptive drive via peripheral afferent fibers was available to maintain a state of central sensitization. Still, we also observed 5-HT3 receptors, shown previously to be expressed on spinal neurons^40^, to be necessary for nociceptive sensitization, glial activation and augmented chemokine expression. The CXCR2 receptors targeted by CXCL1 and CXCL2 to support sensitization were previously demonstrated to be expressed on spinal neurons^14^. Thus post-TBI nociceptive sensitization may involve descending 5-HT3 signaling and a neuron-glia-neuron circuit similar to that which supports sensitization after peripheral injuries, but with descending 5-HT release rather than peripheral input maintaining the sensitized state.

Though we present evidence for dual pathways of spinal sensitization after TBI, there are limitations to this work. For example, the present work is primarily focused on the early period after TBI, yet it is not clear whether contributions from these mechanisms persist into the chronic period. Loss of descending inhibition (as opposed to facilitation) may be the more relevant phenomenon in chronic pain after TBI^10,12,31^. We also lack clinical data at this point evaluating descending pain modulation in TBI patients directly using robust psychophysical measurements, although such techniques are available^41^. Translational efforts such as these are critical to advancing the hypothesis that endogenous pain modulatory mechanisms regulate the pain experience after TBI.

## Materials and Methods

### Animals

Male C57Bl/6J mice (Jackson Laboratories; Bar Harbor, MA, U.S.A) were acclimated to our animal facility for at least 7 days upon arrival before beginning of experimentation. The mice were 12 weeks old at time of experiments and were kept under standard vivarium conditions of 12 h light/dark cycle and ambient temperature. Prior to initiation of experiments, mice were habituated to handling by the experimenters that were blind to the identity of planned treatments or experimental conditions. The Veterans Affairs Palo Alto Health Care System Institutional Animal Care and Use Committee (Palo Alto, CA, USA) approved all experimental procedures and protocols in accordance with the guidelines of National Institutes of Health Guide for the Care and Use of Laboratory Animals.

### Closed-head model of mild traumatic brain injury (mTBI)

The closed head mTBI and sham procedures were based on previously established protocols and described in our prior publication^13,42^. Briefly, a benchmark stereotaxic impactor (MyNeurolab, St. Louis, MO, USA) actuator was mounted on a stereotaxic frame (David Kopf Instruments, Tujunga, CA, USA) at a 40° angle with a 5-mm impactor tip. After isoflurane anesthesia induction, mice were placed in a foam mold, held in prone position on the stereotaxic frame, and maintained under anesthesia for the duration of the procedure. The stereotaxic arm was adjusted so that the head impact was at a fixed point relative to the right eye and ear, corresponding to the S1 somatosensory cortex. The impact delivered by the device to the head was 5.8–6.0 m/s with a dwell time of 0.2 s and impact depth of 5 mm. After impact, the mice were recovered from anesthesia on a warming pad prior to returning to their home cages. Skull fractures were not observed in our study similar to previously reported studies using comparable head impact forces^42,43^. For sham groups, the above procedure was performed except that the impact device was discharged in the air.

### Mechanical nociceptive assay

Mechanical sensitivity was assessed as described previously^13^ using nylon von Frey filaments (Stoelting Co., IL, USA) according to the “up-down” algorithm developed by Chaplan *et al*.^44^. We have applied this technique previously to estimate withdrawal thresholds in mice after injury^45,46^. After acclimating mice on the wire mesh platform inside plastic enclosures (10 cm radius), sequential fibers with increasing stiffness were applied to the plantar surface of hind limb and left in place for 5 sec. When 4 fibers had been applied after the first response the testing was terminated. Withdrawal of hind paw from the fiber was considered a response. If a response occurred after application of a fiber then a less stiff fiber was applied, if no response was observed the next stiffest fiber was applied. Mechanical withdrawal threshold was determined by a data fitting algorithm to allow for determination of significance using parametric statistical analysis^47^.

### Drug treatments

In order to assess the role of serotonin signaling in TBI-induced mechanical hypersensitivity, either systemic or spinal 5-hydroxytryptamine/serotonin (5-HT) depletion was performed. For systemic 5-HT depletion, mice were treated with p-Chlorophenylalanine dissolved in saline (PCPA, 100 mg/kg, *i.p*., Sigma-Aldrich, MO, USA). PCPA inhibits the enzyme tryptophan hydroxylase, a rate-limiting enzyme in the biosynthesis of 5-HT, that results in a functional 5-HT synthesis blockade. The dose and treatment regimen were chosen to cause major depletion of central 5-HT levels^18,33^. Vehicle animals received saline injections instead of PCPA. The spinal 5-HT depletion experiments were done as described previously^17^, the spinal serotoninergic neurons of the lumbar spinal cord were targeted using an intrathecal injection of 5,7-dihydroxytryptamine diluted in sterile saline (5,7-DHT, 50 µg/10 µl, *i.t*., Adipogen Corp, CA, USA). 5,7-DHT is a neurotoxin that destroys 5-HT axons and nerve terminals when injected into the brain and/or spinal cord. To prevent the partial depletion of norepinephrine that can occur following 5,7-DHT injection mice were pretreated with desipramine hydrochloride diluted in sterile saline (25 mg/kg, *i.p*., Tocris Bioscience, MN, USA) 45-min prior to 5,7-DHT administration. The treatment regimen chosen has been shown to result in a major reduction of dorsal lumbar spinal tissue 5-HT levels within 4 days^19,48^. Vehicle animals received desipramine hydrochloride injections and saline injections instead of 5,7-DHT. Four days post-intrathecal injection, vehicle and 5,7-DHT treated-mice were randomly selected to be given a mTBI or sham procedure.

The next experiment assessed the effect of the selective 5-HT_3_ receptor antagonist, ondansetron diluted in sterile saline (*i.t*., 1 or 3 µg/10 µl; Sigma-Aldrich, USA) on mechanical sensitivity on post-TBI day 3. The doses of ondansetron chosen were based on our and other published reports^34,49^. Vehicle animals received saline injections instead of ondansetron.

The effects of CXCR2 antagonist, SCH527123, (MCE, Medical Express, NJ, U.S.A.) on TBI-induced mechanical hypersensitivity was assessed following systemic (1, 3 and 10 mg/kg, *i.p*. for up to 7 days) or spinal (2.5 µg/5 µl, *i.t*, once) administration Vehicle animals received saline injections instead of SCH527123.

For intrathecal injections, mice were briefly anesthetized with isoflurane and the spinous process of the L6 was located and the vertebrate column around this area was secured by pressing it gently. The 30 G needle was carefully inserted between the groove of L5 and L6 vertebrae and successful entry of the needle into the intradural space was confirmed by the observation of a tail flick. Following the injection, the mouse was recovered using standard procedure.

### Total RNA isolation, reverse transcription and real-time polymerase chain reaction (PCR)

We used previously established and described methods to perform the PCR experiments^14^. The lumbar spinal cord was isolated and divided into contralateral and ipsilateral segments (relative to the mTBI), placed in RNA preservative (RNA later, Qiagen, CA, USA) and then frozen at −80 °C. Later, the contralateral samples were homogenized using a Polytron device (Brinkman Instruments Inc., NY, USA), then centrifuged for 10 min at 12,000 times gravity at 4C. The supernatants were then processed using the RNeasy Mini Kit (Qiagen, CA, USA) according to manufacturer’s instructions. The purity and concentration of the purified RNA was determined spectrophotometrically. Subsequently, cDNA was synthesized from this total RNA using random hexamer priming and a First Strand cDNA Synthesis Kit (Invitrogen, CA, USA). Real-time PCR, reactions were conducted using the Sybr Green I master kit (PE applied Biosystems, Foster City, CA, USA) as described in detail previously^14^. Briefly, a mixture of sybr green and CXCL1, CXCL2, CXCL3, CXCL5, CXCL7 or CXCR2 primers was loaded with diluted cDNA template in each well. Melting curves were performed to document single product formation, and agarose electrophoresis confirmed product size. 18 s RNA (Ambion, Austin, TX, USA) was used as an internal control. The data from real-time PCR experiments were analyzed by the comparative CT (2^−∆∆Ct^) method after normalizing to 18 s RNA from the same cDNA preparations.

### Protein isolation

On post-TBI days 3 or 7, the lumbar spinal cords were collected (L3-5) and split into pieces contralateral and ipsilateral to the mTBI. Both pieces of the lumbar spinal cord were separately placed in a phosphate buffered solution (PBS) containing Protein kinase inhibitor (Roche Applied Science, USA), and frozen at −80 °C for further analysis. The contralateral side of the lumbar spinal cord was then homogenized using an ultrasonic approach. After centrifugation of the homogenates for 15 min at 12,000 g, 4 °C, the supernatants were aliquoted and protein contents were measured using the DC Protein Assay kit (Bio-Rad, USA).

### ELISA assay

CXCL1, CXCL2 and CXCL3 levels were determined in duplicate by using respective EIA kits, according to the manufacturer’s instructions. The ELISA kits were obtained from R&D Systems. These assay systems detect mouse CXCL1, CXCL2 and CXCL3 with sensitivity of 1.3 pg/ml. The concentrations of the proteins were calculated from the standard curve at each assay. Each protein concentration was expressed as pg/mg total protein, as described previously^14^.

### Immunohistochemistry

After transcardially perfusing mice with ice-cold saline followed by 4% ice-cold paraformaldehyde, lumbar spinal cords were post-fixed in 4% paraformaldehyde and then cryoprotected in 20% sucrose in phosphate buffered saline (PBS) 4 °C. We then followed previously described methods to process the samples and perform image analysis^17^. Spinal cords (from the L3 to L5 segments) were cut into 20 µm transverse sections using a cryostat. Immunostaining was performed using rabbit anti-glial fibrillary acidic protein for astrocytes (GFAP; 1:1000, AB5804, Millipore-Sigma) and rabbit anti-Ionized calcium binding adaptor molecule-1 for microglia (IBA-1, 1:750, 019–19741, Wako, VA, USA). Both antibodies were visualized using the 3,3′-diaminobenzidine (DAB) method so sections were pre-treated with 0.3% hydrogen peroxide in PBS for 30 minutes prior to immunostaining. Blocking of the all sections took place at room temperature for 2 hours in PBS containing 10% normal goat serum (Vector Laboratories, CA, USA), followed by exposure to the primary antibodies for 24 hours at 4 °C. After rinsing they were visualized, using biotin-conjugated second antibodies (Vector Laboratories), followed by incubation with the Vectastain Elite ABC reagent (Vector Laboratories) and developed using the DAB peroxidase substrate kit (Vector Laboratories). Controls prepared with primary antibody omitted showed minimal background DAB staining under the conditions employed. Staining was performed concurrently for each group of sections compared with one another and imaged under identical conditions.

### Image analysis

In all data assessments, both the image collection and analysis were performed by observers blinded to experimental conditions. GFAP, and IBA-1 expression was evaluated bilaterally in the spinal cord; 5 non-consecutive sections, spaced 200 μm apart and spanning L4 were selected. Sections from uninjured, untreated mice were used to establish a threshold level that excluded all non-specific staining. This threshold level was then applied to all experimental groups. The percentage of the total image area covered by GFAP, and IBA-1 in each section was calculated using the “area fraction” feature in the NIH ImageJ program.

### Data analysis

Mechanical sensitivity data were analyzed by multiple t-tests with Holm-Sidak method to correct for multiple comparisons (no assumption of same scatter). Random groups of mice were assigned to undergo drug or vehicle treatments and accessed till recovery. Consequently, there were measurements for 10–11 time points for TBI with systemic and spinal 5HT depletion, 4 timepoints for ondansetron experiments, 3 timepoints for systemic CXCR2 antagonist and 6 timepoints for spinal CXCR2 antagonist administration. One-way ANOVA and Fisher’s LSD multiple comparisons tests were used for gene expression studies, protein analysis and immunohistochemistry data analysis. Data are presented as mean ± S.E.M and for all analyses and significance determined as p < 0.05.

## Data Availability

The datasets generated during and/or analyzed during the current study are available from the corresponding author on reasonable request.
